# Supplementary material for: Stabilization of Calcium Oxalate Precursors during the Pre- and Post-Nucleation Stages with Poly(acrylic acid)
Source: Nanomaterials (Basel). 2021 Jan 18;11(1):235. doi: 10.3390/nano11010235 (PMC7829898; doi:10.3390/nano11010235)
Supplement: Supplementary file 1 [file nanomaterials-11-00235-s001.pdf]

SUPPLEMENTARY MATERIAL

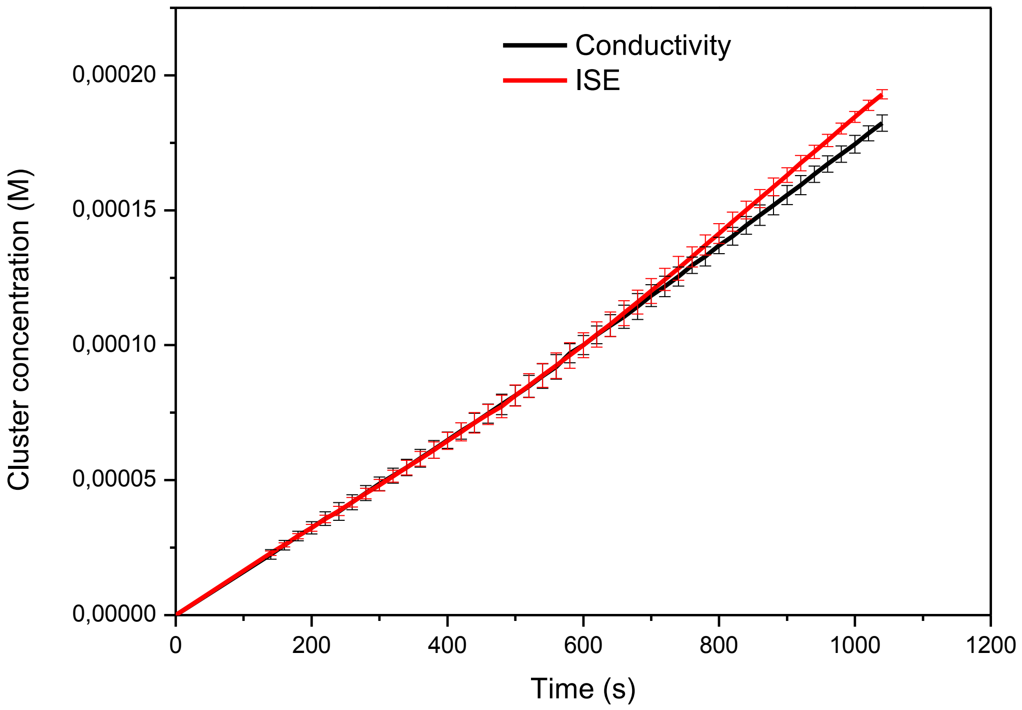

Figure S1. Time development of pre-nucleation cluster concentration using continuous conductivity and ion selective electrode (ISE) measurements. The curves show a perfect match of the time evolution of ionic complexes concentration based on a 2 : 1 Ca:C_2O_4, binding ratio, as determined by the conductivity monitoring of ions concentration in solution (black line) and from ISE (red line).

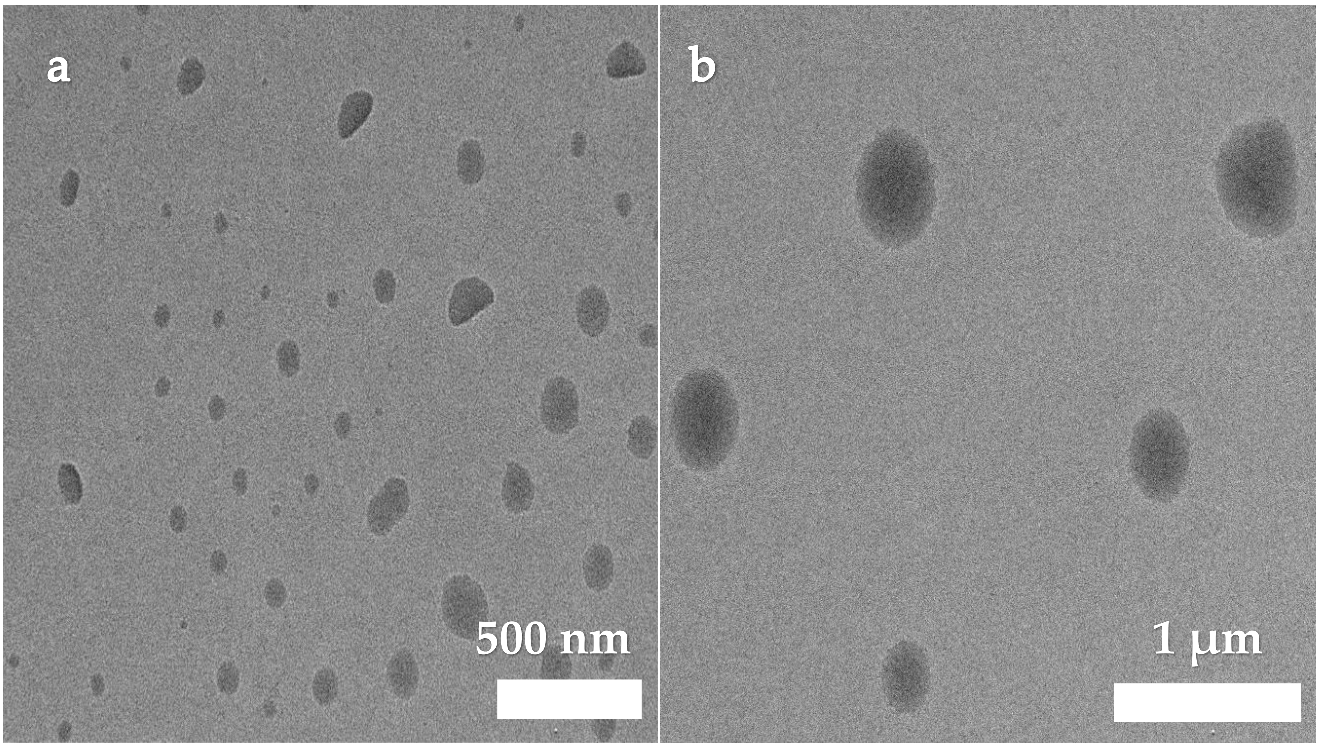

Figure S2. TEM images of ACO nanoparticles during the early stages of nucleation in the presence of PAA 100 mg/L.

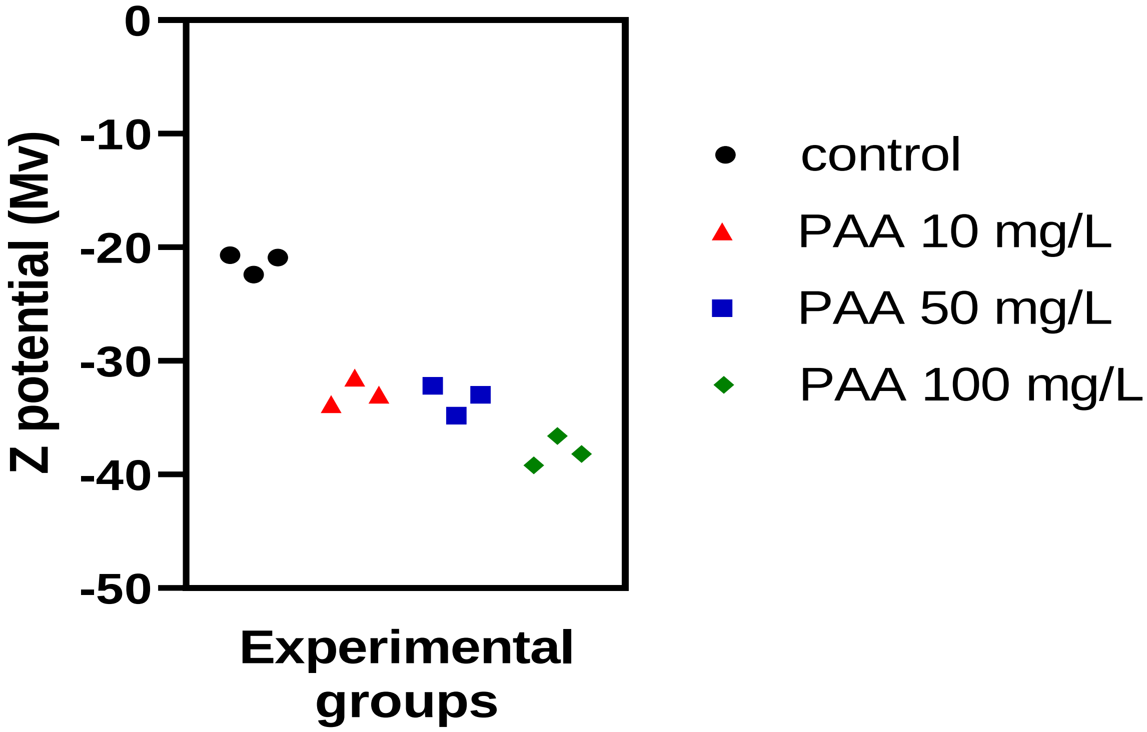

Figure S3. Zeta potential of nanoparticles obtained during the pre-nucleation stage in the absence of PAA and in the presence of three concentrations of PAA.

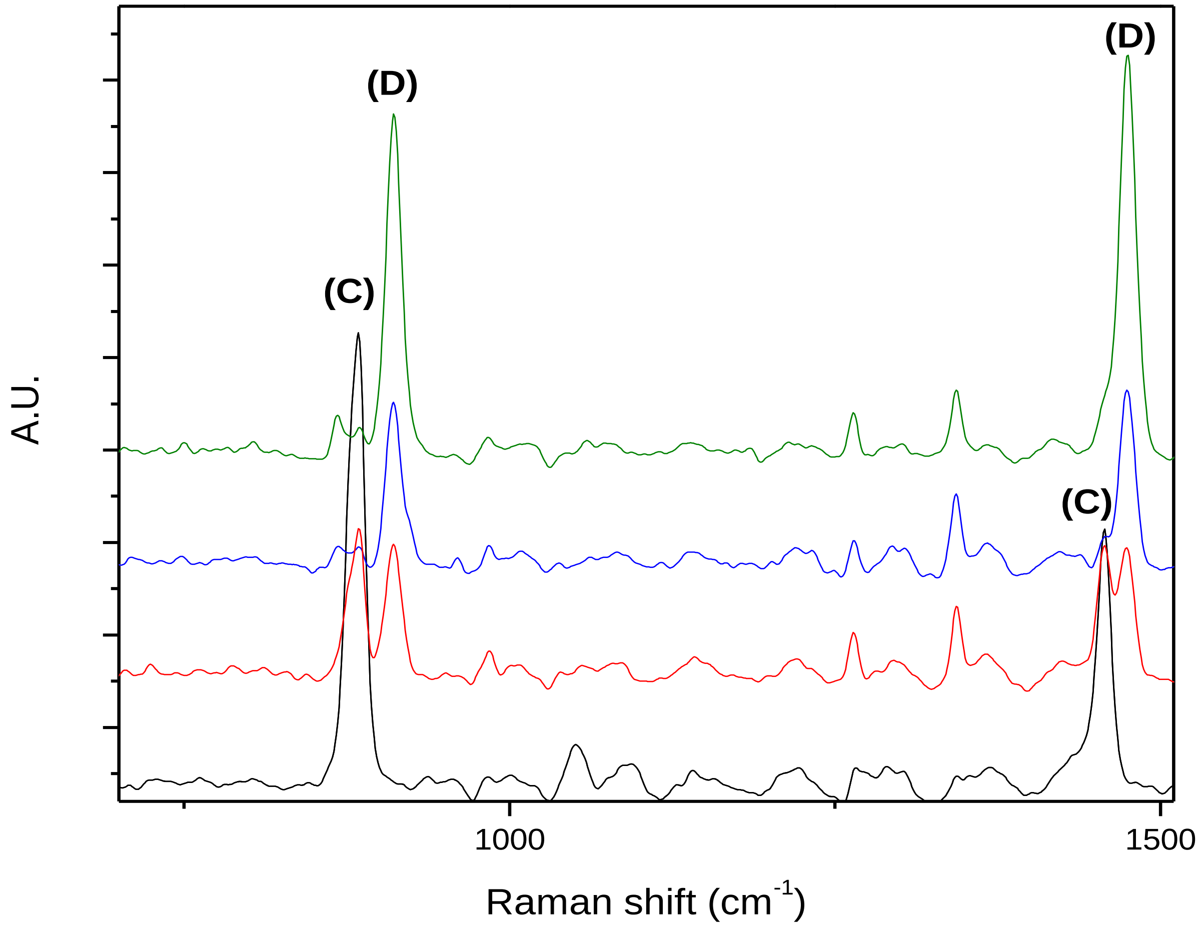

Figure S4. Raman spectroscopy of CaOx particles obtained during the titration experiment in the absence (control) and in the presence of PAA in the post-nucleation stage. The black line indicates the control experiment run and the red, blue and dark green lines indicate the runs performed in the presence of 10, 50 and 100 mg/L PAA, respectively. The abbreviations of C and D indicate COM and COD, respectively.

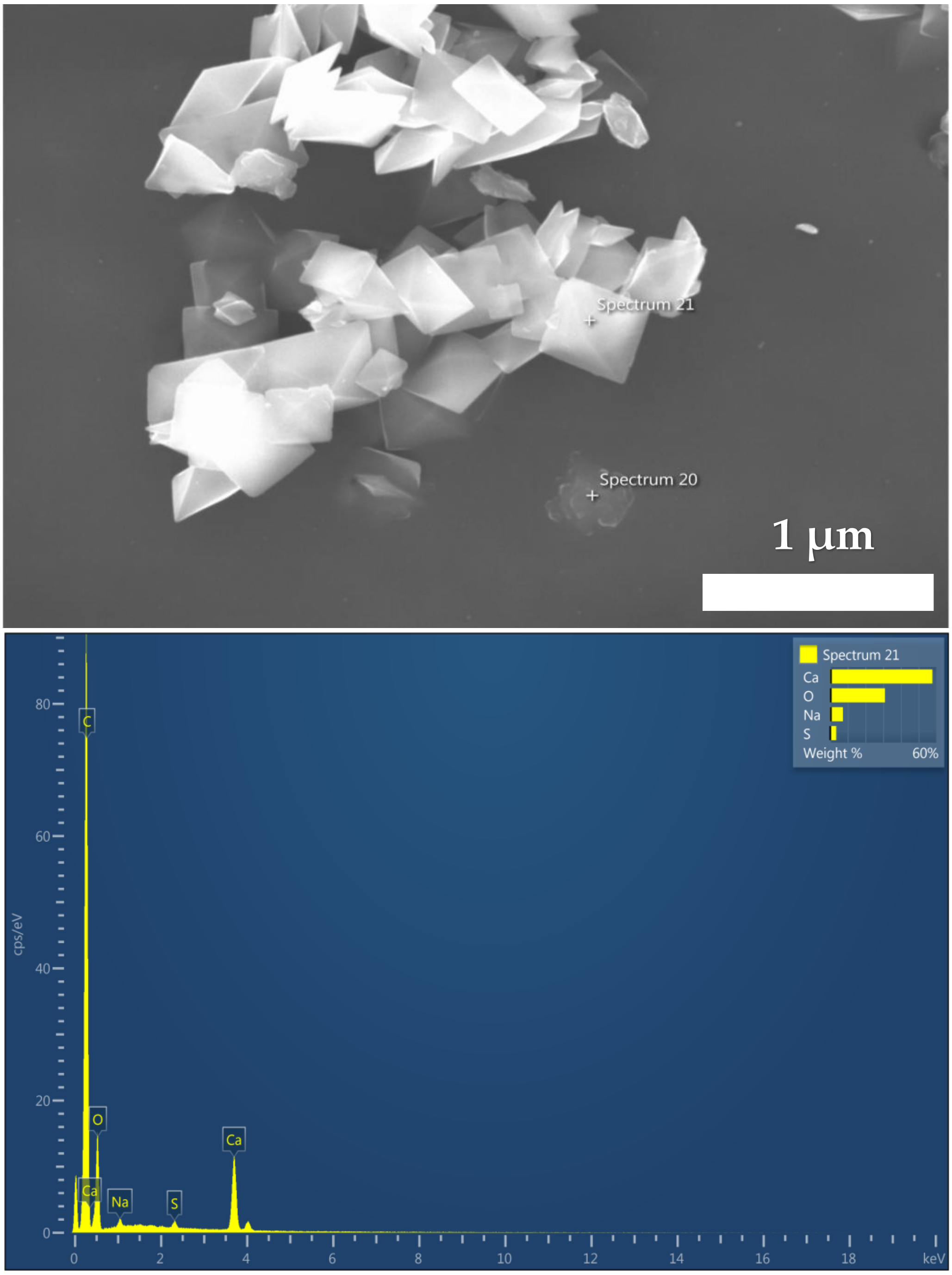

Figure S5. FESEM with EDS analysis of CaOx precipitates obtained during the post-nucleation stage in the presence of PAA 100 mg/L, showing pseudo-dipyramidal crystals probably ACO nanoparticles and large pyramidal crystals.

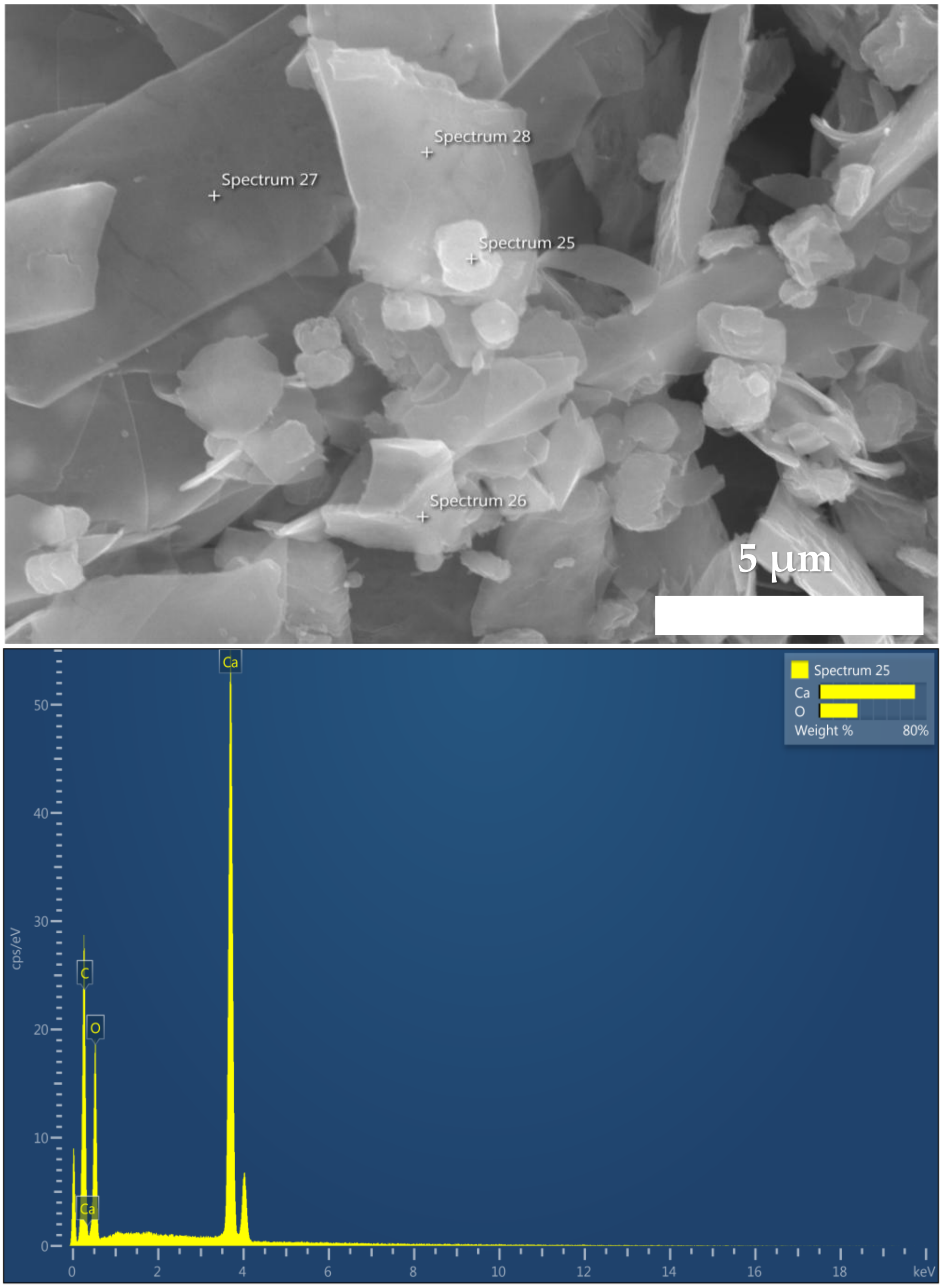

Figure S6. FESEM with EDS analysis of CaOx precipitates obtained during the post-nucleation stage in the presence of PAA 10 mg/L, showing spheroidal or pseudo-dipyramidal crystals probably ACO nanoparticles and large particles with typical shape corresponding to COD and COT.
